# *Francisella tularensis* 2-C-Methyl-D-Erythritol 4-Phosphate Cytidylyltransferase: Kinetic Characterization and Phosphoregulation

**DOI:** 10.1371/journal.pone.0020884

**Published:** 2011-06-09

**Authors:** Arthur Tsang, Heather Seidle, Safdar Jawaid, Weidong Zhou, Clint Smith, Robin D. Couch

**Affiliations:** 1 Department of Chemistry and Biochemistry, George Mason University, Manassas, Virginia, United States of America; 2 National Center for Biodefense and Infectious Diseases, George Mason University, Manassas, Virginia, United States of America; 3 Department of Molecular and Microbiology, George Mason University, Manassas, Virginia, United States of America; 4 Center for Applied Proteomics and Molecular Medicine, George Mason University, Manassas, Virginia, United States of America; 5 Geospatial Research and Engineering Division, U.S. Army Engineer Research and Development Center, Alexandria, Virginia, United States of America; University of Louisville, United States of America

## Abstract

Deliberate and natural outbreaks of infectious disease, the prevalence of antibiotic resistant strains, and the ease by which antibiotic resistant bacteria can be intentionally engineered all underscore the necessity of effective vaccines and continued development of novel antimicrobial/antiviral therapeutics. Isoprenes, a group of molecules fundamentally involved in a variety of crucial biological functions, are derived from either the mevalonic acid (MVA) or methylerythritol phosphate (MEP) pathway. While mammals utilize the MVA pathway, many bacteria utilize the MEP pathway, highlighting the latter as an attractive target for antibiotic development. In this report we describe the cloning and characterization of *Francisella tularensis* MEP cytidylyltransferase, a MEP pathway enzyme and potential target for antibiotic development. Size exclusion chromatography indicates the protein exists as a dimer in solution. Enzyme assays produced an apparent 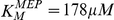, 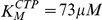, 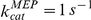, 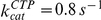, and a 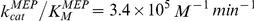. The enzyme exhibits a strict preference for Mg^+2^ as a divalent cation and CTP as the nucleotide. Titanium dioxide chromatography-tandem mass spectrometry identified Thr141 as a site of phosphorylation. T141D and T141E site-directed mutants are catalytically inactive, suggesting a mechanism for post-translational control of metabolic flux through the *F. tularensis* MEP pathway. Overall, our study suggests that MEP cytidylyltransferase is an excellent target for the development of novel antibiotics against *F. tularensis*.

## Introduction

Biothreat agents are classified by the US Centers for Disease Control and Prevention (CDC) based upon their morbidity/mortality rates, ease of dissemination, emergency response procedures, and projected social impact. Category A and B biothreat agents are of greatest concern and include *Bacillus anthracis* (the causative agent of anthrax), *Yersinia pestis* (plague), *Francisella tularensis* (tularemia), *Brucella* species (brucellosis), *Burkholderia mallei* (glanders), and *Burkholderia pseudomallei* (melioidosis). Our vulnerability to both deliberate and natural outbreaks of infectious disease is illustrated by the 2009 H1N1 swine flu pandemic, 2003 SARS outbreak, 2001 anthrax letter attacks, and 1984 Rajneeshee *Salmonella* attacks, highlighting the necessity of effective vaccines and antimicrobial/antiviral therapeutics. The prevalence of antibiotic resistant strains and the ease by which antibiotic resistance can be deliberately engineered into bacteria further underscores the need for continued development of novel antibiotics against new bacterial targets.

**Figure 1:**
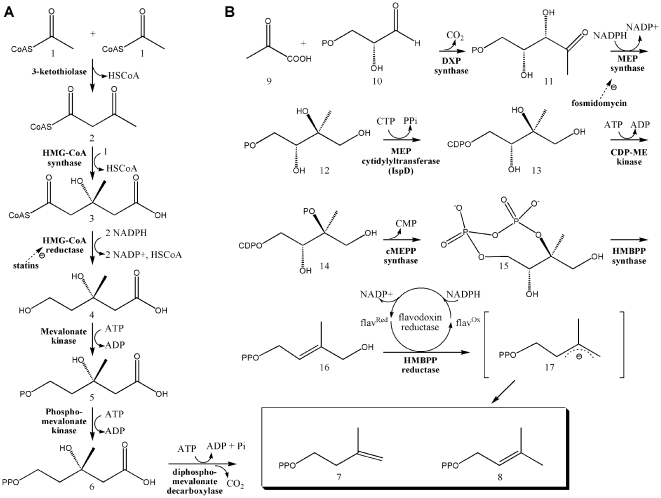
Two pathways of isoprene biosynthesis. A) The MVA Pathway. Three molecules of acetyl-CoA (1) are condensed to form 3-hydroxy-3-methylglutaryl CoA (HMG-CoA) (3), which is then reduced to MVA by HMG-CoA reductase (4) [20], [21]. MVA is further processed to yield IPP (7) [22]–[24], which is converted to DMAPP (8) by an isomerase [25]. B) The MEP Pathway. Pyruvate (9) is condensed with glyceraldehyde 3-phosphate (10) to yield 1-deoxy-D-xylulose 5-phosphate (DXP; (11)) [26], an intermediate with a role in *E. coli* vitamin B1 and B6 biosynthesis [27]–[30] as well as isoprene biosynthesis. 1-Deoxy-D-xylulose 5-phosphate reductoisomerase (also called MEP synthase) catalyzes the reduction and rearrangement of 11 to yield MEP (12) [31], the first committed step in the *E. coli* MEP pathway. The next enzyme, MEP cytidylyltransferase (or IspD), converts MEP into 4-(cytidine 5′-diphospho)-2-C-methyl-D-erythritol (CDP-ME; (13)). CDP-ME kinase then phosphorylates CDP-ME, which becomes cyclized (coupled with the loss of CMP) by cMEPP synthase to yield 2-C-methyl-D-erythritol 2,4-cyclodiphosphate (15) [14], [32]–[35]. A reductive ring opening of 15 produces 1-hydroxy-2-methyl-2-butenyl diphosphate (HMBPP; (16)) [36]–[40], which is reduced to both IPP and DMAP in a ∼5∶1 ratio [4], [41]–[46].

Antibiotic development generally proceeds through five distinguishable phases, including target identification, target validation, lead molecule identification, lead molecule optimization, and preclinical and clinical trials [1]. Comparative genomic investigations, utilizing bioinformatics and complete genome sequences, has greatly empowered the target identification phase of novel antimicrobial drug development [2]. Genome sequences reveal that *Francisella*, *Brucella*, *Bacillus*, *Burkholderia*, and *Yersinia* each harbor methylerythritol phosphate (MEP) pathway genes involved in isoprene biosynthesis. Isoprenoids are a large and structurally diverse class of molecules essential to a variety of crucial biological functions, including cell wall biosynthesis (dolichols), the regulation of membrane fluidity (hopanoids and cholesterol), electron transport (quinones), and signal transduction (prenylated proteins). Despite their diversity, all isoprenoids are synthesized from two building blocks, isopentenyl diphosphate (IPP) and dimethylallyl diphosphate (DMAPP), which are produced by the mevalonic acid (MVA) or MEP pathway (Fig. 1). Because the MVA pathway is exclusively utilized by mammals, the bacterial MEP pathway enzymes are promising targets for the development of novel antibiotics (reviewed in [3]–[5]).

Target validation typically involves loss of function assays (e.g. small molecule inhibition and/or genetic knockout) to determine the essentiality of the target, followed by protein characterization to ascertain its amenability to high-throughput screening (HTS) assays (HTS is often used in the lead identification phase, wherein large molecular libraries are screened for inhibitors of the target protein). Growth inhibition by fosmidomycin [6] (a specific inhibitor of MEP synthase) and lethal genetic knockouts of MEP synthase (FTN_1483) and MEP cytidylyltransferase (FTN_0623) [7] illustrate the vulnerability of *F. tularensis* to MEP pathway inhibition. We recently detailed the enzymatic characterization of *F. tularensis* MEP synthase and demonstrated its amenability to high-throughput inhibitor screens [6]. We describe here the cloning and enzymatic characterization of *F. tularensis* MEP cytidylyltransferase and report on its tractability to HTS assays. We detail the apparent Michaelis-Menten kinetic constants, survey the cation and nucleotide specificity of the enzyme, and assess its quaternary structure in solution. Additionally, we use titanium dioxide chromatography-tandem mass spectrometry to identify a site of phosphorylation on the enzyme, and suggest that phosphoinhibition may serve to regulate metabolic flux through the MEP pathway. Overall, our results validate MEP cytidylyltransferase as a promising target for the development of novel antibiotics against *F. tularensis*.

## Materials and Methods

### Bacterial strains and growth conditions

*Francisella tularensis* subsp. *holarctica* Strain LVS (FSC 155) was obtained through the NIH Biodefense and Emerging Infections Research Resources Repository, NIAID, NIH, and was cultured at 37°C in tryptic soy broth supplemented with 0.1% cysteine. *Escherichia coli* BL21 CodonPlus (DE3)-RIL cells (Stratagene, La Jolla, CA) were used for recombinant protein expression whereas *E. coli* TOP10 (Invitrogen, Carlsbad, CA) or XL1Blue (Stratagene) cells were used for general cloning procedures. *E. coli* was grown at 37°C in Luria-Bertani (LB) media with constant shaking (250 rpm). Protein expression was performed in LB media containing ampicillin (100 µg/ml) and chloramphenicol (50 µg/ml). Solid media were prepared by addition of 1.5% (wt/vol) agar.

### Genomic and Plasmid DNA isolation

Genomic DNA was isolated from *F. tularensis* subsp. *holarctica* using a Wizard Genomic DNA Purification Kit (Promega, Madison, WI), per the manufacturer's instructions. A GenElute Plasmid miniprep kit (Sigma-Aldrich, St. Louis, MO) was used to isolate plasmid DNA from *E. coli*.

### Construction of the *F. tularensis* MEP cytidylyltransferase expression plasmid

The MEP cytidylyltransferase coding region (*ispD,* FTL_1525) was identified in the *F. tularensis* subsp. *holarctica* LVS genome (accession number NC_007880) via a BLAST search using the *E. coli* K12 homologous sequence as the query. Polymerase chain reaction (PCR) primer pairs, designed to flank *ispD* (FtIspD-f; 5′- CACCGTGTCAAATAAATACGTAATTATTC -3′ and FtIspD-r; 5′- ACCTAACTGCC AATTAGCATATTC -3′), were purchased from Sigma-Genosys (The Woodlands, TX) and used to amplify the gene from purified genomic DNA. The primer FtIspD-f contains four additional 5′ residues (CACC) to facilitate the unidirectional insertion of the PCR product into plasmid pET101/D-TOPO (Invitrogen). FtIspD-r is designed to eliminate the stop codon in the PCR product to permit the expression of a C-terminal His-tagged MEP cytidylyltransferase protein. PCR was performed with Platinum Pfx polymerase (Invitrogen) and the following parameters: 2 minutes at 94°C followed by 30 cycles of 30 sec at 94°C, 30 sec at 53°C, 1.5 min at 68°C, and a final elongation of 10 min at 68°C. A Qiaquick PCR Purification Kit (Qiagen, Valencia, CA) was used to purify the PCR product, which was then cloned into pET101/D-TOPO to create the plasmid pFtIspD. Restriction mapping and DNA sequencing were used to confirm the fidelity of the PCR and the correct construction of the plasmid. pFtIspD was transformed into chemically competent *E. coli* BL21 CodonPlus (DE3)-RIL cells to express the protein.

### Expression and purification of *F. tularensis* MEP cytidylyltransferase

Shake flask cultures (1 L) were used for protein expression (37°C, 250 rpm). A 10 mL overnight culture of *E. coli* BL21 CodonPlus (DE3)-RIL containing pFtIspD was used to inoculate the shake flask and upon the cell density reaching an OD_600_ of 1.1, protein expression was induced by the addition of 0.5 mM isopropyl β-D-thiogalactopyranoside (IPTG). After incubation for an additional 18 hours, cells were harvested by centrifugation and stored at −80°C. To purify the His-tagged protein, the cell pellet was thawed then cells were lysed using Lysis Buffer A (100 mM Tris pH 8, .032% lysozyme; 3 mL per mg cell pellet), followed by Lysis Buffer B (0.1 M CaCl_2_, 0.1 M MgCl_2_, 0.1 M NaCl, .020% DNase; 0.3 mL per mg cell pellet). Clarified cell lysate was obtained by centrifugation (48,000 x g, 20 min) then passed through a TALON immobilized metal affinity chromatography column (Clontech Laboratories, Mountain View, CA). The column was washed with 15 column volumes of 1X equilibration buffer (50 mM HEPES pH 7.5, 300 mM NaCl), 10 column volumes of 1X wash buffer (50 mM HEPES pH 7.5, 300 mM NaCl, 10 mM imidazole) and the His-tagged protein was then eluted with 4 column volumes of 1X elution buffer (150 mM imidazole pH 7.0, 300 mM NaCl). Buffer was exchanged by addition of 0.1 M Tris pH 7.5, 1 mM NaCl while concentrating the protein by ultrafiltration. Protein concentration was determined using the Advanced Protein Assay Reagent (Cytoskeleton, Denver, CO) with γ-globulins (Sigma-Aldrich) as the standard. The protein was visualized via Coomassie stained SDS-PAGE and a Western blot with an anti-His antibody (Qiagen). The yield of purified MEP cytidylyltransferase averaged 20–30 mg per 1 L LB shake flask.

### Mutagenesis

The Thr_141_ to Asp_141_ and Thr_141_ to Glu_141_ mutants of MEP cytidylyltransferase were engineered by GenScript Corporation (Piscataway, NJ) using the pFtIspD plasmid as the template for mutagenesis. The sequence of both mutant constructs were confirmed by nucleotide sequencing. The mutant genes (in pET101/D-TOPO) were transformed into chemically competent *E. coli* BL21 CodonPlus (DE3)-RIL cells to express the C-terminal His-tagged protein. The T141D and T141E mutants were purified as described for the wildtype His-tagged MEP cytidylyltransferase.

### Size-Exclusion Chromatography

The molecular mass of MEP cytidylyltransferase and the mutant derivatives were estimated by loading 1 mg of protein onto a Sephacryl 200HR (Sigma Aldrich, St. Louis, MO) size-exclusion chromatography column equilibrated with 0.1 M Tris pH 7.5, 1 mM NaCl (flow rate of 2 mL/min) and calibrated with a gel filtration standard kit purchased from Bio-Rad (Hercules, CA). Blue dextran was used to determine the void volume of the column.

### Fluorescence spectroscopy

Fluorescence spectra of wildtype and mutant MEP cytidylyltransferase were measured using a Fluoromax-3 fluorometer (Horiba Jobin Yvon) at an excitation wavelength of 290 nm using cuvettes with an optical path length of 1 cm. The emission spectra of protein samples with a concentration of 5 µM in 0.1 M Tris pH 7.5, 1 mM NaCl were measured from 310 to 400 nm (excitation and emission slit width = 5 nm). The temperature was maintained at 30°C. All fluorescence spectra were corrected for background with pure buffer.

### Enzyme Assays

MEP cytidylyltransferase activity was evaluated using a coupled-enzyme colorimetric assay, derived from that originally described by Bernal *et al* [8]. To determine the apparent K_M_ for 2-C-methyl-D-erythritol 4-phosphate (MEP), the assay (200 µL) was performed using 2.5 µM IspD, 100 mM Tris pH 8.0, 1 mM MgCl_2_, 1 mM DTT, 0.2 mM CTP, 100 mU/mL inorganic pyrophosphatase, and varying concentrations of MEP (Echelon Biosciences, Salt Lake City, UT). The assay mixture was incubated at 37°C while 40 µL aliquots were removed at 30 sec intervals and combined with 120 µL water and 40 µL of an acidic malachite green solution (2.4 M sulfuric acid containing 1.5% ammonium molybdate, 1.6% Tween 20, and 9.5% malachite green). After a 10 minute incubation, sodium citrate was added to 3.4%. After an additional 30 min incubation the A_660_ was spectrophotometrically determined. A pyrophosphate standard curve was used to calculate the rate of the enzyme. To determine the apparent *K*_M_ for CTP, the assay was performed as described but with a fixed concentration of MEP (400 µM) and a variable CTP concentration. To determine cation specificity, assays were performed with 0.2 mM MEP, 0.2 mM CTP, and 1 mM MgCl_2_, CaCl_2_, CoCl_2_, CuCl_2_, MnCl_2_, or ZnCl_2_. Nonlinear regression fitting to the Michaelis-Menten equation was used to determine the kinetic constants. High-throughput assays were performed using 96-well plates with assay volumes adjusted to 50 µL. The Z-factor was calculated as described by Zhang *et al* [9] with calcium as the inhibitor control.

### Mass spectrometry method for phosphopeptide identification

To obtain MEP cytidylyltransferase for phosphopeptide analysis, protein expression and purification was essentially as described above, with the exception of using 0.01 mM IPTG for induction. Purified MEP cytidylyltransferase was reduced with 10 mM DTT), alkylated by iodoacetamide (50 mM), and then digested by trypsin (Promega) in buffer containing ammonium bicarbonate (50 mM, pH 9) and urea (2 M). The digestion mixture was then desalted by a SepPak column (Waters, Milford, MA). Phosphopeptides were enriched from the tryptic peptides by a TiO_2_ column as previously described [6]. The purified phosphopeptides were analyzed by reversed-phase liquid chromatography nanospray tandem mass spectrometry (LC-MS/MS) using an LTQ-Orbitrap mass spectrometer (ThermoFisher) using previously described methods [10]. Tandem mass spectra were searched using the program SEQUEST (Bioworks software, Thermo) with full tryptic cleavage constraints, static cysteine alkylation by iodoacetamide, and variable phosphorylation of Ser/Thr/Tyr. Phosphopeptide identification was determined using database match scoring criteria filters followed by manual evaluation of the raw data, as described [10].

### Molecular Modeling

The *F. tularensis* subsp. *holarctica* LVS MEP cytidylyltransferase (protein accession number CAJ79964) was homology-modeled using I-TASSER (http://zhanglab.ccmb.med.umich.edu/I-TASSER/) [11]. Templates were selected by I-TASSER's threading alignment algorithm which considers predicted secondary structure features in the sequence and identifies analogous and homologous protein templates. The optimized model was evaluated with ProQRes [12], which uses atom-atom contacts, residue-residue contacts, solvent accessibility, and secondary structure information to assign an accuracy score from 0 (unreliable) to 1 (reliable). Swiss-PdbViewer 4.0 (http://spdbv.vital-it.ch/) was used to visualize and annotate the model.

## Results and Discussion

### Cloning, expression, and purification of *F. tularensis* MEP cytidylyltransferase

**Figure 2:**
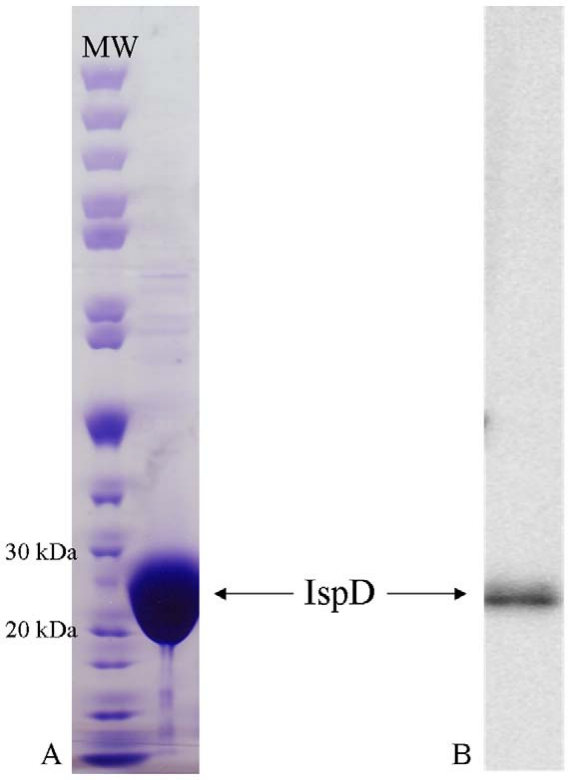
Purification of recombinant *F. tularensis* MEP cytidylyltransferase. A) Coomassie stained SDS-PAGE showing a molecular weight marker (MW) and purified His-tagged MEP cytidylyltransferase. His-tagged MEP cytidylyltransferase has a predicted molecular weight of 29.5 kDa. B) Western blot hybridization analysis of purified MEP synthase using an anti-His antibody results in an intense band of the expected size.

The 690 bp *ispD* gene (FTL_1525) from *F. tularensis* subsp. *holarctica* LVS encodes a polypeptide of 229 amino acids with a calculated molecular mass of 25.9 kDa. The subsp. *holarctica* amino acid sequence shares 99.1, 98.3, and 99.6% identity with the MEP cytidylyltransferase sequence from subsp. *tularensis* (FTT_0711), subsp. *novicida* (FTN_0623), and subsp. *mediasiatica* (FTM_1371), respectively. PCR was used to amplify subsp. *holarctica ispD* and the resulting product was cloned into an expression plasmid designed to express a C-terminal His-tagged protein in *E. coli*. The recombinant protein was purified by immobilized metal affinity chromatography and the protein was visualized by SDS-PAGE and Western blotting using an anti-His antibody (Fig. 2). Size-exclusion chromatography using a calibrated column indicates that the *F. tularensis* MEP cytidylyltransferase exists in solution as a dimer (Fig. S1), similar to the MEP cytidylyltransferase from *Mycobacterium tuberculosis* [13] and *E. coli* [14], [15].

### Kinetic characterization of *F. tularensis* MEP cytidylyltransferase

**Figure 3:**
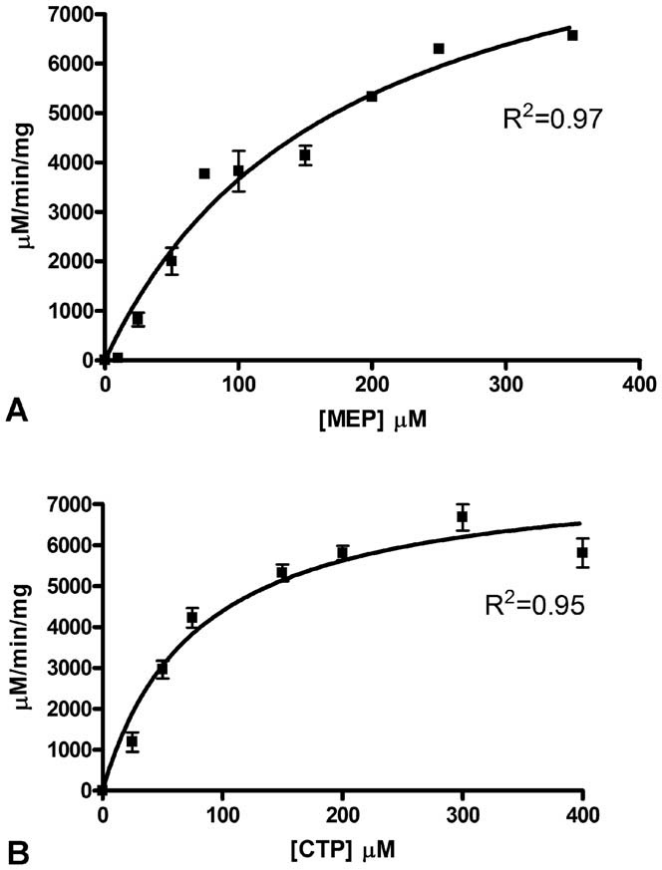
Substrate dependent activity of *F. tularensis* MEP cytidylyltransferase. Michaelis-Menten plot of reaction velocity as a function of A) MEP concentration and B) CTP concentration. The solid line represents the nonlinear least-squares best fit of the data to the Michaelis-Menten equation. Each assay was performed in duplicate. The R^2^ value for each plot is indicated. Kinetic parameters derived from the plots are listed in Table 1.

**Figure 4:**
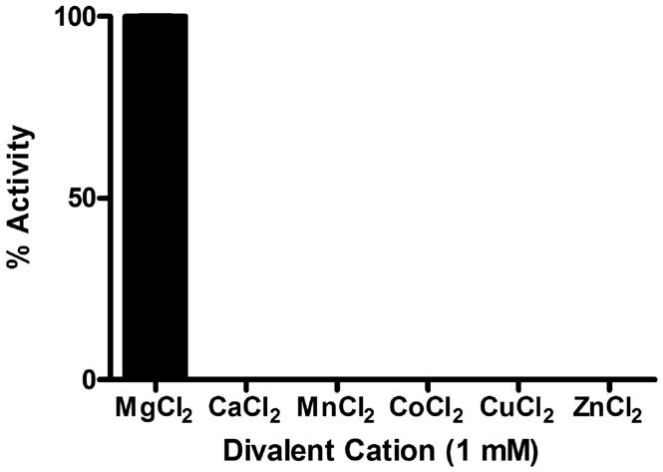
The divalent cation specificity of *F. tularensis* MEP cytidylyltransferase. Enzyme assays were performed with the indicated divalent cations at a fixed MEP (200 µM) and CTP (200 µM) concentration. Relative enzyme activity reveals the strict preference of the enzyme for Mg^+2^. Each assay was performed in duplicate.

**Figure 5:**
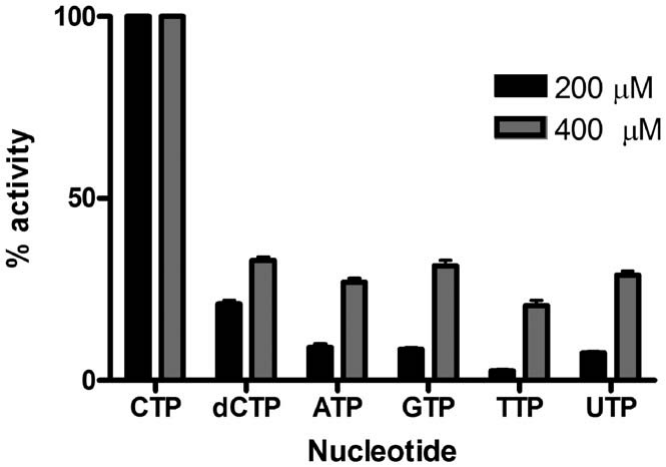
The nucleotide specificity of *F. tularensis* MEP cytidylyltransferase. Enzyme assays were performed with the identified nucleotide at either 200 µM or 400 µM, as indicated. The assays contained a fixed MEP (200 µM) concentration. Each assay was performed in duplicate. Relative enzyme activity reveals the preference of the enzyme for CTP.

The kinetic activity of purified MEP cytidylyltransferase was spectrophotometrically evaluated by monitoring the substrate dependent enzyme catalyzed production of pyrophosphate (Fig. 1B). Nonlinear regression fitting of enzyme velocity versus substrate concentration was used to determine the apparent kinetic constants (Fig. 3 and Table 1). The 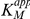 for MEP was obtained using assays performed with a saturating concentration of CTP (200 µM), whereas the 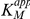 for CTP was determined using assays with saturating levels of MEP (400 µM). Overall, the 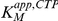 for the *F. tularensis* MEP cytidylyltransferase is similar to the reported values for the *E. coli* and *M. tuberculosis* orthologs, when assayed accordingly (Table 1), while the 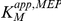 is considerably larger for the *F. tularensis* enzyme. Additionally, the catalytic turnover 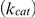 of the *E. coli* and *F. tularensis* enzymes are comparable, while each are slightly depressed relative to the *M. tuberculosis* ortholog (Table 1). Combined with differences in apparent 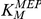 values, the disparity between the turnover numbers results in considerably different specificity constants 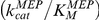 for the *F. tularensis* and *M. tuberculosis* enzymes. The biological significance of this remains unclear. Interestingly, cation specificity assays reveal a strict preference of the *F. tularensis* MEP cytidylyltransferase for MgCl_2_ (Fig. 4), unlike the *E. coli* and *Mycobacterium* orthologs which also accommodate Mn^+2^ or Co^+2^ [13], [14]. Additionally, the *F. tularensis* MEP cytidylyltransferase displays a clear preference for CTP over alternative nucleotides (Fig. 5).

**Table 1:** MEP cytidylyltransferase Apparent Kinetic Parameters.

MEP cytidylyltransferase	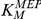 (µM)	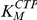 (µM)	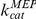 (s^-1^)	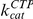 (s^-1^)	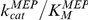 (M^-1^min^-1^)	Refer.
*F. tularensis*	177.9 +/- 35.2	73.0 +/- 14.9	1.0 +/- 0.10	0.8 +/- 0.05	3.4×10^5^ +/- 6.7×10^4^	This study^a^
*E. coli*	61 +/- 14 370 +/- 60	58 +/- 6 760 +/- 60	0.7 25.9 +/- 5.9	- 54.1 +/- 28.0	6.6×10^5^ 7.86×10^6^	[8] a [18] b
*M. tuberculosis*	43 58.5 +/- 5.4	92 53.2 +/- 4.5	3.4 0.012	- 0.017	4.7×10^6^ 1.23×10^4^	[13] a [47] c

a Colorimetric assay using malachite green.

b Radiometric assay using ^14^C-MEP.

c Radiometric assay using γ-^32^P-CTP.

### Phosphorylation of *F. tularensis* MEP cytidylyltransferase

**Figure 6:**
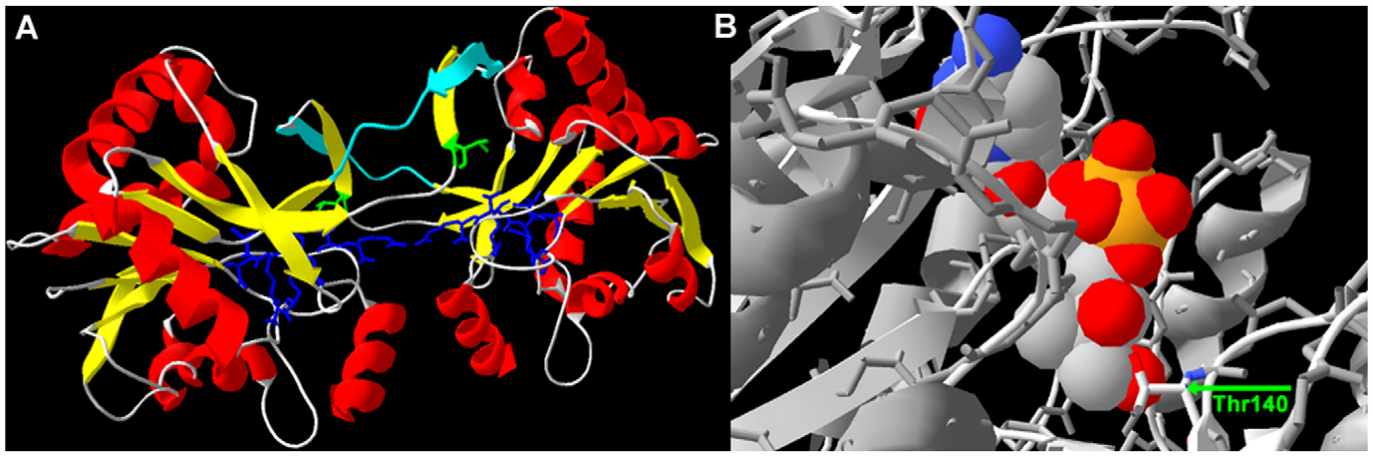
Structural features of the MEP cytidylyltransferase. A) Predicted structure of the *F. tularensis* MEP cytidylyltransferase, homology-modeled using templates selected by I-TASSER's threading alignment algorithm. A cartoon representation of the dimeric enzyme is shown, with alpha helices colored red, beta sheets colored yellow, and coiled regions colored gray. ProQRes was used to evaluate the quality of the model (Fig. S2), providing scores ranging from 0 (unreliable) to 1 (reliable). Regions of the model scoring <0.5 are colored light blue. Residues comprising the substrate binding site (colored dark blue with backbone and sidechain residues shown) were identified via primary sequence alignment and the resolved structure of *E. coli* MEP cytidylyltransferase [15]. Threonine141 (colored green with backbone and sidechain residues shown) is equivalent to *E. coli* Thr140. B) Active site of the *E. coli* MEP cytidylyltransferase co-crystallized with CDP-ME (PDB accession code 1INI). Thr140 (arrow) hydrogen bonds to the C1 and C3 hydroxyl oxygen atoms of CDP-ME [15], [18].

Multifarious control mechanisms converge to collectively regulate the activity of HMG-CoA reductase, thereby controlling metabolic flux through the MVA pathway (reviewed in [16]). In contrast, engineered expression studies suggest that several pathway enzymes share control of regulating flux through the MEP pathway (reviewed in [17]). We recently described the phosphorylation of *F. tularensis* MEP synthase, illustrating its potential for metabolic flux control and highlighting a region of the protein that may be amenable to rational drug design [6]. To ascertain if the *F. tularensis* MEP cytidylyltransferase might also be phosphoregulated, we expressed the recombinant enzyme in *E. coli* (induced with 10 µM IPTG), purified the His-tagged protein, subjected the protein to trypsinization, and isolated and identified phosphopeptides via titanium dioxide chromatography-tandem mass spectrometry. Phosphothreonine 141 (equivalent to threonine 140 in the *E. coli* homolog) was identified (Fig. 6a). The resolved crystal structure of *E. coli* MEP cytidylyltransferase in complex with CDP-ME (Fig. 6b) shows Thr140 positioned in the substrate binding site, with the sidechain hydroxyl and backbone amide groups hydrogen bonded to the C3 and C1 hydroxyl oxygen atoms of ME, respectively [15], [18]. Threonine 141 in the *F. tularensis* homolog presumably has a similar role.

**Figure 7:**
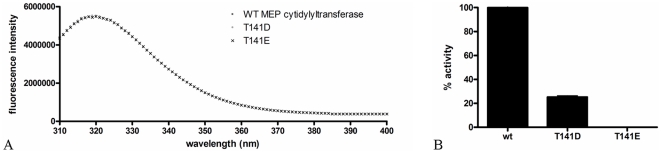
Regulation of *F. tularensis* MEP cytidylyltransferase. A) Intrinsic fluorescence spectra of MEP cytidylyltransferase and two mutant derivatives. Wildtype and mutant (T141D and T141E) proteins were adjusted to 5 µM in 0.1 M Tris pH 7.5, 1 mM NaCl and analyzed using an excitation wavelength of 290 nm. The emission spectra was measured from 310 to 400 nm. The Em λ_max_ of all three proteins was detected at 320 nm. Identical spectra indicate little structural change in the protein globular fold accompanies the introduction of either Asp or Glu. B) The relative activity of wildtype MEP cytidylyltransferase and the T141D and T141E mutants. Assays were performed in duplicate with 200 µM MEP and 200 µM CTP.

In light of the above observations, we hypothesized that phosphorylation of Thr141 would disrupt the enzyme-substrate interaction, negatively affecting enzyme activity. To test this hypothesis, two mutants of *F. tularensis* MEP cytidylyltransferase were created, T141D and T141E, wherein Thr141 was changed to an aspartate or glutamate, respectively, which serve to mimic a phosphothreonine. Each mutant was expressed in *E. coli* and purified to near homogeneity via a C-terminal His-tag. Relative to the wildtype enzyme, little difference was observed in the intrinsic fluorescence maximum (Em λ_max_) for either of the mutants (Fig. 7a), indicating that the introduction of the Asp or Glu has little structural influence on the protein globular fold. Size-exclusion chromatography indicates that both mutants exist as dimers in solution, as was observed with the wildtype isoform (Fig. S1). Enzyme assays using the purified proteins reveal that 75% of wildtype activity is lost with the T141D mutation, whereas the T141E mutant is completely inactive (Fig. 7b). Collectively, these results suggest that phosphorylation of Thr141 in *F. tularensis* MEP cytidylyltransferase inhibits the enzyme, possibly serving as a control mechanism for regulating metabolic flux through the MEP pathway (analogous to the phosphoregulation of HMG-CoA reductase [19], a kinase/phosphatase cycle may control metabolic flux by phosphoregulating the activity of MEP synthase and MEP cytidylyltransferase). Additionally, these results highlight Thr141 for the rational design of small molecule inhibitors of MEP cytidylyltransferase.

### *F. tularensis* MEP cytidylyltransferase and drug discovery

Lead molecule identification by high-throughput inhibition assays typically involves the screening of large molecular libraries using multi-well plate assays, a process that is highly dependent on the quality and robustness of the assay, often described in terms of the Z-factor [9]. An excellent high-throughput assay has a Z-factor score between 0.5 and 1.0. To determine the Z-factor for the *F. tularensis* MEP cytidylyltransferase assay, we adjusted the assay volume to accommodate a 96-well plate, fixed the substrate concentration at the *K_m_* (178 µM), used a saturating concentration of CTP (200 µM), and evaluated three separate lots of purified MEP cytidylyltransferase in a series of assays performed over three consecutive days. Calcium, an inhibitor of pyrophosphatase, was used as a positive control for inhibition. The Z-factor with *F. tularensis* MEP cytidylyltransferase was found to be 0.8, indicative of an assay (and enzyme) well suited for use in a high-throughput screen.

### Conclusions

The target validation phase of novel antibiotic drug development typically involves genetic knockout assays to assess lethality as well as protein purification and characterization to evaluate amenability to HTS and/or rational drug design. While transposon mutagenesis demonstrated the essentiality of MEP cytidylyltransferase to *F. tularensis* viability, the protein remained to be characterized. We demonstrate here that purified recombinant *F. tularensis* MEP cytidylyltransferase is kinetically active and highly amenable to use in HTS. Additionally, our investigation is the first to show that MEP cytidylyltransferase may be phosphoregulated at Thr141, further implicating a phosphoregulatory mechanism of controlling metabolic flux through the *F. tularensis* MEP pathway, as well as highlighting the residue for the rational design of enzyme inhibitors.

## Supporting Information

Figure S1 **Molecular weight determination by size-exclusion chromatography.** Protein standards (▪) were used to calibrate the column. Linear regression fitting (R^2^ is indicated) generated the standard curve, which was used to determine the molecular weight of wildtype MEP cytidylyltransferase (IspD) and the two mutants (T141D and T141E).
(TIF)

Figure S2 **ProQRes evaluation of the** ***F. tularensis*** **MEP cytidylyltransferase structural model generated by I-TASSER.** ProQRes uses atom-atom contacts, residue-residue contacts, solvent accessibility, and secondary structure information to score the model over a sliding window of 9 residues [12]. Scores range from 0 (unreliable) to 1 (reliable).
(TIF)
