# Supplementary figures and images for: Francisella tularensis 2-C-Methyl-D-Erythritol 4-Phosphate Cytidylyltransferase: Kinetic Characterization and Phosphoregulation
Source: PLoS One. 2011 Jun 9;6(6):e20884. doi: 10.1371/journal.pone.0020884 (PMC3111433; doi:10.1371/journal.pone.0020884)

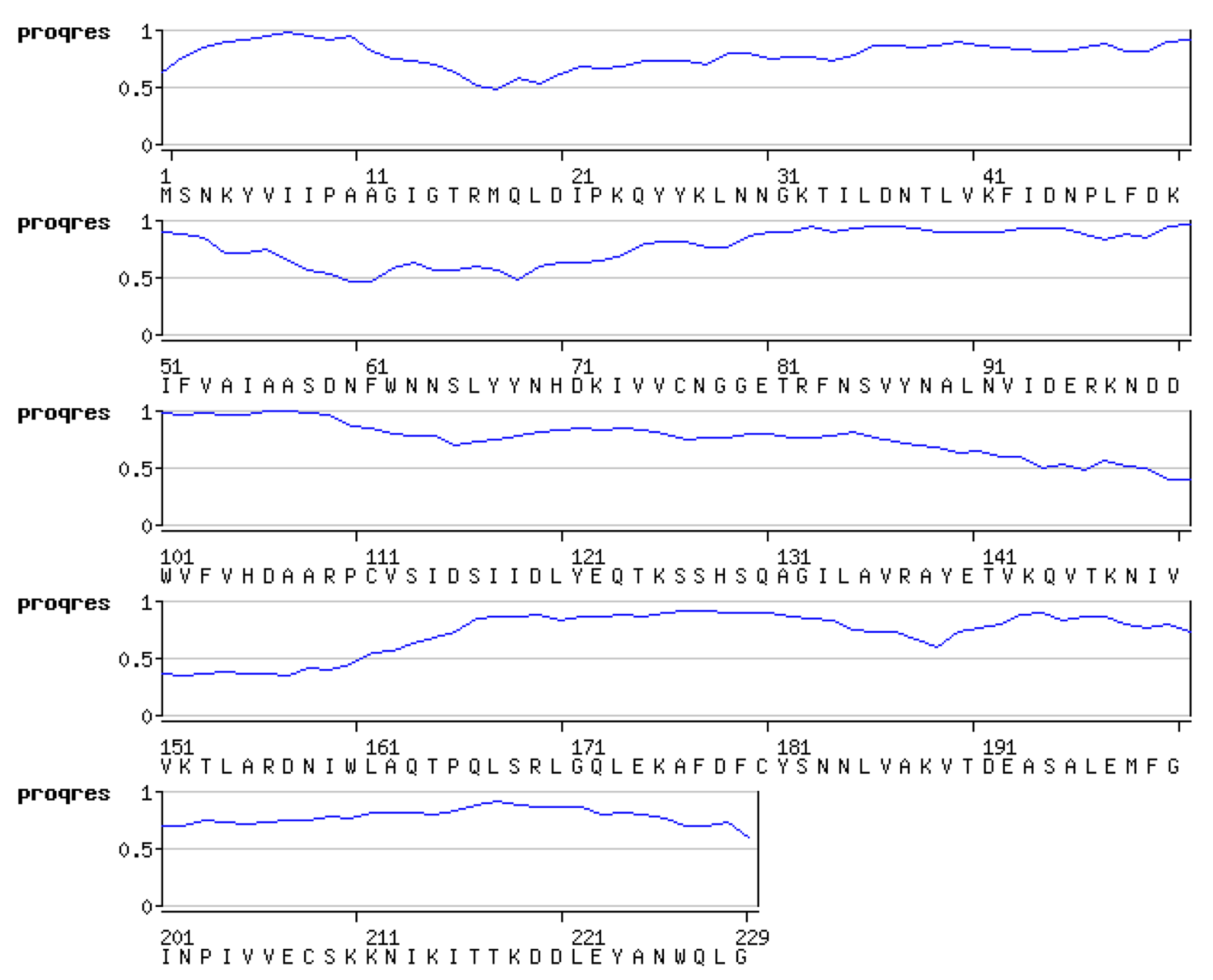

Supplement: Figure S2 — ProQRes evaluation of the F. tularensis MEP cytidylyltransferase structural model generated by I-TASSER. ProQRes uses atom-atom contacts, residue-residue contacts, solvent accessibility, and secondary structure information to score the model over a sliding window of 9 residues [12]. Scores range from 0 (unreliable) to 1 (reliable). (TIF) [file pone.0020884.s002.tif]
